# Fate and Phytotoxicity of CeO_2_ Nanoparticles on Lettuce Cultured in the Potting Soil Environment

**DOI:** 10.1371/journal.pone.0134261

**Published:** 2015-08-28

**Authors:** Xin Gui, Zhiyong Zhang, Shutong Liu, Yuhui Ma, Peng Zhang, Xiao He, Yuanyuan Li, Jing Zhang, Huafen Li, Yukui Rui, Liming Liu, Weidong Cao

**Affiliations:** 1 College of Resources and Environmental Sciences, China Agricultural University, Beijing, 100093, China; 2 Key Laboratory for Biomedical Effects of Nanomaterials and Nanosafety, Institute of High Energy Physics, Chinese Academy of Sciences, Beijing, 100049, China; 3 Key Laboratory of Nuclear Analytical Techniques, Institute of High Energy Physics, Chinese Academy of Sciences, Beijing, 100049, China; 4 Beijing Synchrotron Radiation Facility, Institute of High Energy Physics, Chinese Academy of Sciences, Beijing, 100049, China; 5 Stockbridge School of Agriculture, University of Massachusetts, Amherst, Massachusetts, 01003, United States of America; 6 Institute of Agricultural Resource and Regional Planning, Chinese Academy of Agricultural Science, Beijing, 100081, China; Banaras Hindu University, INDIA

## Abstract

Cerium oxide nanoparticles (CeO_2_ NPs) have been shown to have significant interactions in plants. Previous study reported the specific-species phytotoxicity of CeO_2_ NPs by lettuce (*Lactuca sativa*), but their physiological impacts and vivo biotransformation are not yet well understood, especially in relative realistic environment. Butterhead lettuce were germinated and grown in potting soil for 30 days cultivation with treatments of 0, 50, 100, 1000 mg CeO_2_ NPs per kg soil. Results showed that lettuce in 100 mg·kg^-1^ treated groups grew significantly faster than others, but significantly increased nitrate content. The lower concentrations treatment had no impact on plant growth, compared with the control. However, the higher concentration treatment significantly deterred plant growth and biomass production. The stress response of lettuce plants, such as Superoxide dismutase (SOD), Peroxidase (POD), Malondialdehyde(MDA) activity was disrupted by 1000 mg·kg^-1^ CeO_2_ NPs treatment. In addition, the presence of Ce (III) in the roots of butterhead lettuce explained the reason of CeO_2_ NPs phytotoxicity. These findings demonstrate CeO_2_ NPs modification of nutritional quality, antioxidant defense system, the possible transfer into the food chain and biotransformation in vivo.

## Introduction

Cerium oxide nanoparticles (CeO_2_ NPs), as a typical rare earth metal oxide NPs, are commonly used as a polishing agent for ophthalmic lenses, television tubes and glass mirrors and as fuel additives in order to decrease the emissions of particulates.[1–3] As they can enter soil through atmosphere deposition or from biosolids treated in waste water treatment,[4,5] concerns over potential health and environmental risks of CeO_2_ NPs exposure have been raised.[6] CeO_2_ NPs are one of the selected nanomaterial for priority testing by the Working Party of Manufactured Nanomaterials of the Organization for Economic Cooperation and Development (OECD). CeO_2_ NPs are generally recognized as stable and insoluble in biological or environment systems, therefore they can exist for a long time.[7]

Previous reports show that CeO_2_ NPs exist in nanoparticle form and affect physiological processes and molecular response to plants. Zhang et al. systematically studied the uptake, distribution and biotransformation of CeO_2_ NPs in cucumber plants, and verified that Ce presented in the roots as CeO_2_ and CePO_4_ while in the shoots as CeO_2_ and cerium carboxylates.[8,9] Rico et al. showed that CeO_2_ NPs modify the antioxidative stress enzyme activities and macromolecule composition in rice seedlings.[10] Ma et al. demonstrated CeO_2_ NPs could alter expression of glutathione and sulfated metabolic pathways in *Arabidopsis thaliana* (L.) under Petri dish culture condition.[11] The genetoxicity of CeO_2_ NPs on soybean plants was reported by López-Moreno *et al*.[12] Majority of the existed researches were carried out in aqueous[13], sand[14] or agar medium[15], they are generally used as an idealistic environment, without complex components. Soil is a complex system of minerals, organic material, water, gasses, and living organisms. Lee et al. demonstrated that exposure media have significant effects on phytotoxicity of NPs, and suggested that application of NPs in soil is important to understand the terrestrial toxicity of nanoparticles.[16] Zhao et al. found that lipid peroxidation and ion leakage in corn plants were not affected by CeO_2_ NP exposure in soil.[17] The results were different from hydroponic medium condition. Priester et al. found that CeO_2_ NPs could stunt soil-cultivated soybean growth and pod biomass while shut the nitrogen fixation down.[18] However, Wang et al. reported that CeO_2_ NPs had either an inconsequential or a slightly positive effect on plant growth and tomato production cultivated in potting soil at concentrations of 0.1–10 mg·L^-1^.[19] Morales et al. exhibited that CeO_2_ NPs could change the nutritional properties of cilantro.[20] These inconsistencies may be caused by the differences in exposure methods, plant species, and especially culture media.

Lettuce is an edible plant and among the 10 species recommended by US EPA (1996) for phytotoxicity assessment. We previously reported that CeO_2_ NPs significantly inhibited root elongation of lettuce in both aqueous suspension and plant agar medium. Compared to deionized water, the bioavailability of CeO_2_ NPs in the agar medium was reduced, but the sensitivity of asparagus lettuce to CeO_2_ NPs was increased. [15]^,^[21,22] The aim of this work was to evaluate the influence of CeO_2_ NPs on the growth of lettuce plants in soil medium. Representative parameters such as biomasses, chlorophyll, protein, nitrate and soluble sugar contents, antioxidant enzyme activities, and lipid peroxidation were investigated to understand the plant’s defense and response to abiotic stress caused by CeO_2_ NPs. In addition, CeO_2_ NPS uptake and transformation in lettuce plants were studied using ICP-MS and X-ray absorption near edge structure.

## Materials and Methods

### Plant culture and nanoparticle application

The potting soil for this research was obtained from Scotts Miracle-Gro Company and air-dried at room temperature prior to use. Plastic pots with 12 cm in diameter and 10.8 cm in height were filled out with 150 g of potting soil. CeO_2_ NPs (Sigma Aldrich, USA) suspensions were sonicated for 30 min then applied to the soil to obtain final concentrations of 0, 50, 100, 1000 mg NPs per kg soil with 8 replications. Ten seeds of butterhead lettuce (*Lactuca sativa*) were sown at a depth of approximately 1 cm. At 5 d after planting, the seedlings were thinned to 3 plants per pot. The minimum and maximum greenhouse temperature during the test was 26 and 18 C. Each pot was supplied 100 mL deionized water every other day. To avoid interferences with the NPs, no additional fertilizer was added to the pots. After 30 d of growth, chlorophyll contents in the leaves were analyzed using a chlorophyll meter (Konic Minolta SPAD-502 Plus, Japan). Then the plants were harvested.

### Determination of Cerium by ICP-MS

Plant roots and shoots were separated thoroughly washed with deionized water, and lyophilized with a freeze dryer (Alpha 1–2 LD plus, Christ, Germany). The dried tissues were digested with a mixture of concentrated plasma–pure HNO_3_ and H_2_O_2_ (v/v, 4:1) on a heating plate. The obtained residual solutions were then diluted with deionized water and analyzed by ICP-MS (Thermo X7, USA). A standard reference (bush branches and leaves, GBW07602) was also digested and analyzed by ICP-MS to examine the recovery. Indium of 20 ng·mL^−1^ was used as an internal standard to compensate for the matrix suppression and signal drifting. Analytical runs include calibration verification samples, spike recovery samples and duplicate dilutions. The linearity was from 0.1 to 50 ng·mL^−1^, Recovery from GBW07602 was 99%. Spike recovery was 102%. Detection limit was 0.01 ng·mL^−1^.

### Speciation Analysis by X-ray Absorption Near Edge Fine Structure (XANES)

Lyophilized roots of butterhead lettuces were grinded to powder and pressed into slices with a diameter of 10mm and a thickness of 2 mm for XANES analysis. The Ce L_III_ XANES spectra were recorded in fluorescence mode on beamline 1W1B at Beijing Synchrotron Radiation Facility (BSRF). The ring storage energy of the synchrotron radiation accelerator during data collection was 2.5 GeV with current intensity of 50 mA. Cerium phosphate CePO_4_, cerium oxalate Ce_2_ (C_2_O_4_)_3_ and cerium acetate Ce (CH_3_COO) _3_ as well as the three types of CeO_2_ NPs were used as the standard compounds. Data processing and the linear combination fitting (LCF) of the collected spectra were performed in the software program ATHENA. Samples of lettuce treated with the three types of CeO_2_ NPs (1000 mg L^-1^) were also prepared for the XANES analysis.

### Determination of Protein, Nitrate and Soluble Sugar

Fresh leaf and root samples (0.1g) were homogenizing with 1 mL phosphate buffer (50 mM KH_2_PO4 at pH 7.4) by the Mixer Mills (MM400, RETSCH, German), and then centrifuged at 4000×g for 15 min at 4°C.The supernatant were quantified for protein according to the method by Olson et al.[23]

Fresh leaf tissues (0.2 g) were cut in tiny pieces and aced in screw capped tubes to have a water bath at 100°C for 30 min. After cooling, they were centrifuged at 1500*g for 5 min at 4°C. The method of determination nitrate and soluble sugar was conducted following by Cataldo et al and Irigoyen et al.[24,25] Lastly, the absorbance at 625 nm and 410 nm was determined for soluble sugar and nitrate in a UV-visible spectrophotometer (TU-1901, Beijing)

### Stress Response of Butterhead Lettuce to CeO_2_ NPs

Fresh roots and shoots of lettuce separately homogenized with phosphate buffer solution (PBS, 50 mM, pH 7.8) under ice bath, and then centrifuged at speed of 10 000g and 4°C for 10 min. The supernatants were kept for analyses of SOD, POD activities and MDA contents using the assay kits purchased from Nanjing Jiancheng Bioengineering Institute (Nanjing, China).

### Statistical Analysis

The results are expressed as mean ± SE (n = 8). When data are homogeneity of variance, a one-way ANOVA using General Linear Model, followed by Turkey’s Honestly Significant Difference (HSD) test; when data are heterogeneity of variance, Kruskal-Wallis one-way ANOVA be used, These were performed using SPSS statistical package, version 16.0 (SPSS, Chicago, IL). Statistical significance was based on a probability of p≤0.05.

## Results and Discussion

### Ce Contents in Tissues of Lettuce

**Fig 1:**
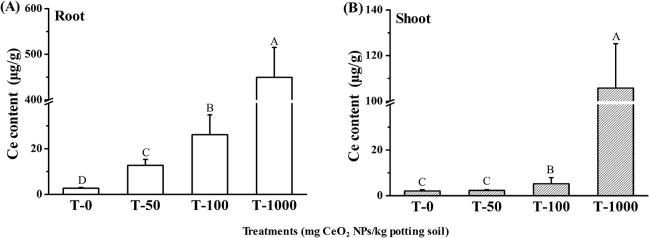
(A) Ce contents in roots (A) and leaves (B) of lettuce plants. Error bars stand for standard errors. Bar with the same letters show no statistically significant differences at p≤0.05. n = 8.

Ce contents in the roots and shoots of 30-day-old lettuce plants treated with CeO2 NPs were determined by ICP-MS. As shown in Fig 1A, the Ce contents in roots increased as the external CeO_2_ NPs increased. From Fig 1B, plants in shoot exposed to 100 mg·kg^-1^ and 1000 mg·kg^-1^ CeO_2_ NPs had significantly higher Ce content compared to control. Ce content in the root (12.7–449.4 μg·g^-1^) was much higher than that in the shoots (2.3–105.8 μg·g^-1^). Compared to previous results[15], Ce concentrations in butterhead lettuce roots and shoots in this study were lower than in agar medium or hydroponic medium. As a consequence, it is difficult to find the CeO_2_ NPs in the roots by TEM.

### Speciation Analysis of Cerium in the Lettuce Plants by XANES

**Fig 2:**
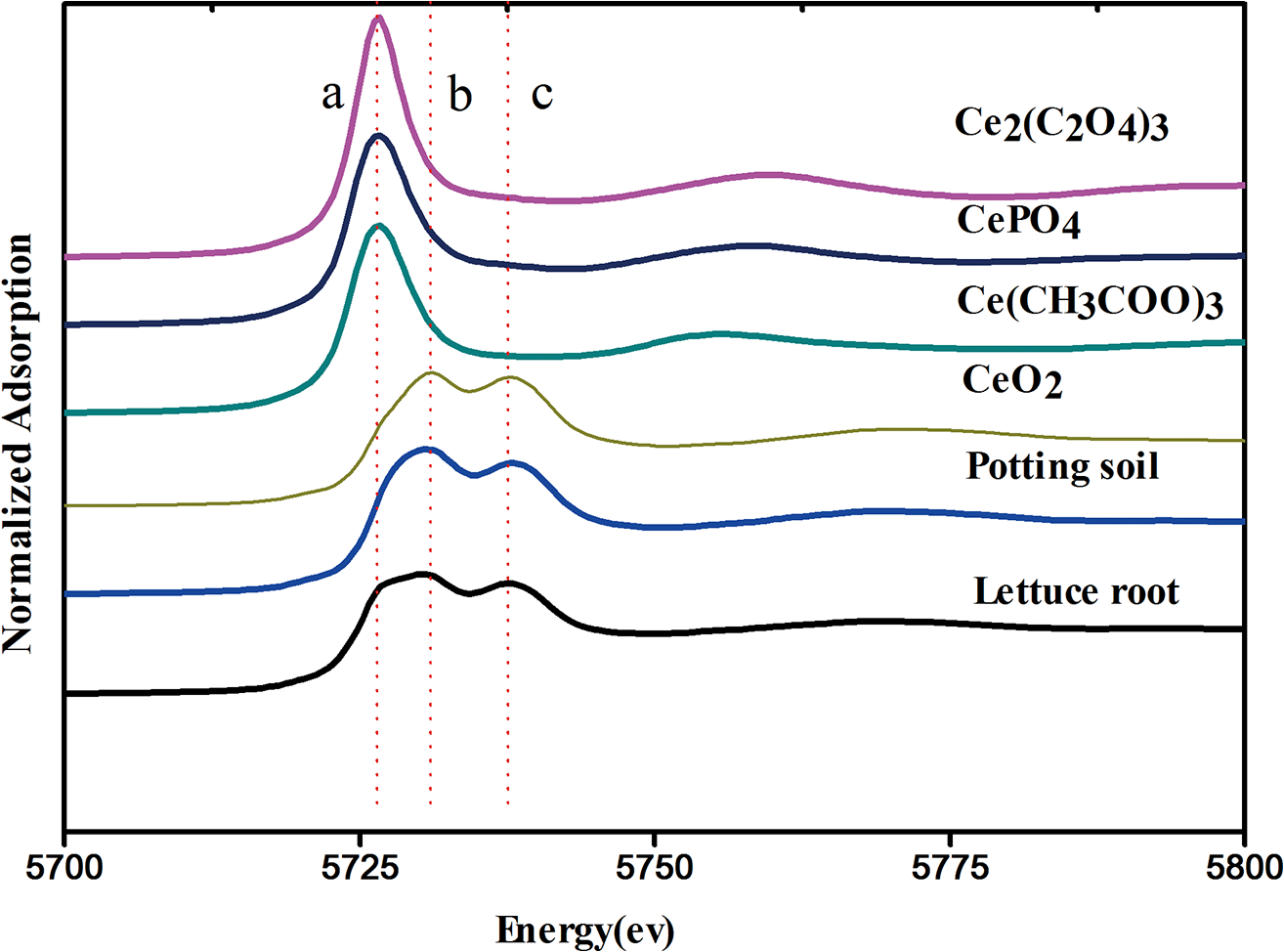
XANES Ce LIII-edge spectra (5723 eV) in roots of butterhead lettuce treated with CeO_2_ NPs. The dotted line indicates the feature a, b and c.

The biotransformation of CeO_2_ NPs determines the ultimate fate and toxicity of manufactured nanoparticles in living organisms. By XANES, the oxidation state of Ce in the butterhead lettuce root can be identified. The XANES spectra of normalized Ce L_III_ edge in the reference Ce compounds and roots of butterhead lettuce are shown in Fig 2. Peak a in the spectra represents the characteristic peak of Ce (III), which come from CePO_4_, Ce (CH_3_COO) _3_ and Ce_2_ (C_2_O_4_)_3_ here. Double peaks B and C indicate the characteristic peak of Ce(IV), which come from CeO_2_ in this study.[26] Compared with standard references, the spectra from roots of treated asparagus lettuce showed the mixed features of peaks A, B and C. These indicate that Ce in roots presents a mixed oxidation state of Ce (IV) and Ce (III). To obtain the quantitative information of Ce species in the roots, LCF was performed on the normalized spectra of samples using CeO_2_, CePO_4_, Ce (CH_3_COO) _3_ and Ce_2_ (C_2_O_4_)_3_ as the standard compounds. The fitted lines and fitting parameters indicate that the results are satisfying and convincing.

The Ce species in the potting soil emerged as 95.1% CeO_2_ and 4.2% Ce_2_ (C_2_O_4_)_3_, without CePO_4_ and Ce (CH_3_COO)_3_. In the root samples, the Ce species presented as 77.3% CeO_2_ and 22.7% Ce carboxylates (included 0.5% CePO_4_, 7.5% Ce (CH_3_COO)_3_ and 12.3% Ce_2_(C_2_O_4_)_3_. According to our previous report[22], Sigma CeO_2_ NPs with non-uniform size contained a large number of small particles less than 25 nm (S1 Fig), so they have higher reactivity and released more Ce^3+^ than the dimension of 25 nm CeO_2_. Particles with smaller size have higher specific surface area and can be expected to show higher reactivity. The release of Ce^3+^ may play a key role in phytotoxicity of metal-based NPs.[27]

### Growth of Lettuce Plants

**Fig 3:**
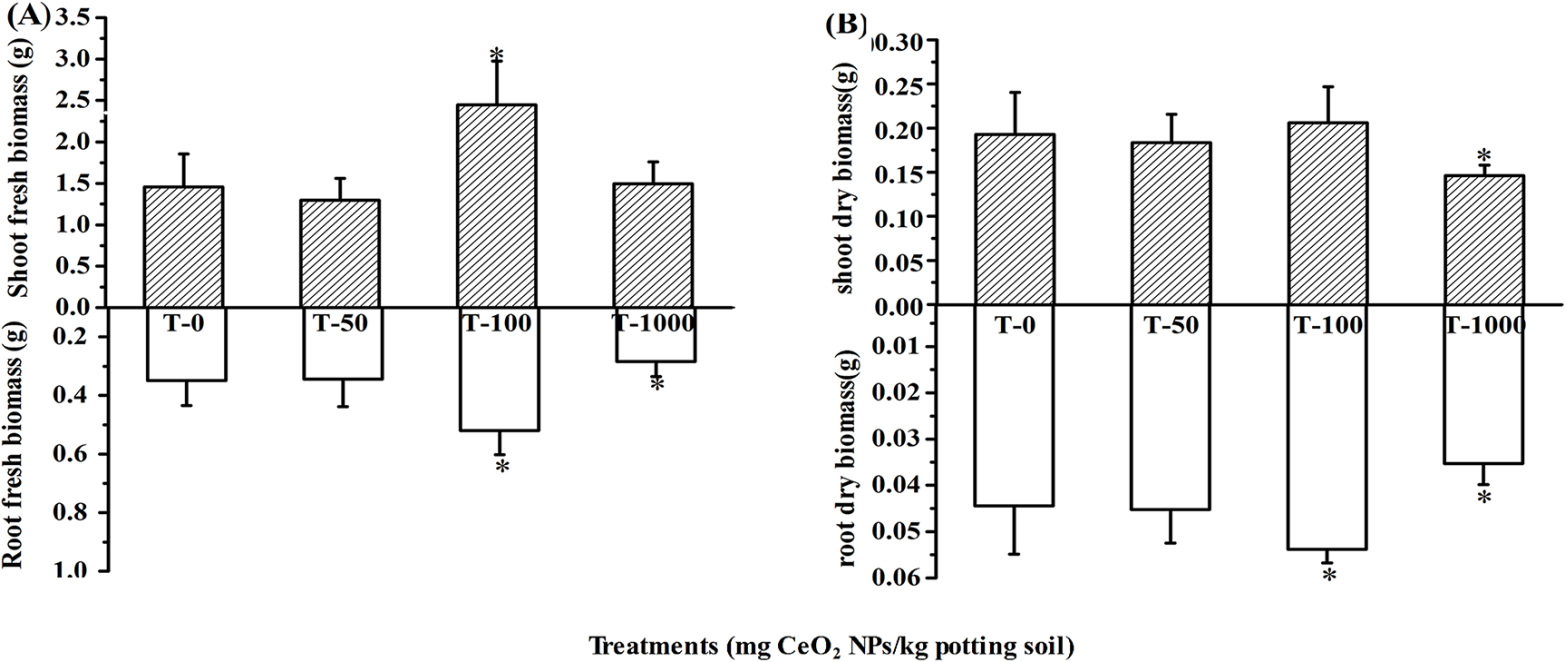
(A) Root and shoot fresh biomass of lettuce plants grown for 30 days in potting soil, treated with 0 (control)-1000 mg·kg^-1^ CeO_2_ NPs. (B) Root and shoot dry biomass of lettuce plants grown for 30 days in potting soil treated with 0 (control)-1000 mg·kg^-1^ CeO_2_ NPs. Error bars stand for standard errors. Bar with this asterisk (*) symbol shows statistically significant differences at p≤0.05. n = 8.

In this study, butterhead lettuce plants were treated with CeO_2_ NPs in potting soil at different concentrations (0, 50, 100, 1000 mg NPs per kg soil). After 30 days, wet and dry mass of the root and shoot of lettuce plants were weighed and shown in Fig 3). It is observed that plants treated with 100 mg·kg^-1^ grew significantly faster than others. The lower concentration (50 mg·kg^-1^) treatment had no impact on plant growth, compared with the control. However, the higher concentration (1000 mg·kg^-1^) treatment significantly decreased the dry masses of root and shoot. The results indicated that CeO_2_ NPs are phytotoxic to plants at a high concentration, and the effect to dry biomass of lettuce plants is more obvious than the fresh ones (more than 94% water in the fresh plant). Rare earth elements (REEs) including cerium can exert positive or negative physiological effects on plants depending on the dosage and other conditions. Much evidence has accumulated in support of the view low concentrations of REEs exert positive effects on growth and yield of crops, whereas high concentrations of REEs seem to be harmful for plants, although the exact mechanism is still unknown.[28] According to our previous results, the effects of CeO_2_ NPs on lettuce plants were probably attributed to the released Ce^3+^ in the roots.

### Chlorophyll and Protein Content

The contents of chlorophyll and protein are important physiological parameters in the development of plant growth. In this study, no significant differences among of treatments were observed, even at a high concentration (1000 mg·kg^-1^) exposure (S2 and S3 Figs) Several studies have demonstrated that ENPs induce modifications on the protein levels in plants at early seedling stage.[16],[29,30] Zhao *et al*. have shown that neither CeO_2_ nor ZnO NPs impacted cucumber chlorophyll content at concentrations of 0, 400, and 800 mg kg^-1^, however, at 800 mg kg^-1^ treatment, CeO_2_ NPs reduced the yield by 31.6%.[31] Different plant species may act differently to the exposure of nanoparticles.

### Nitrate and Soluble Sugar Contents

**Fig 4:**
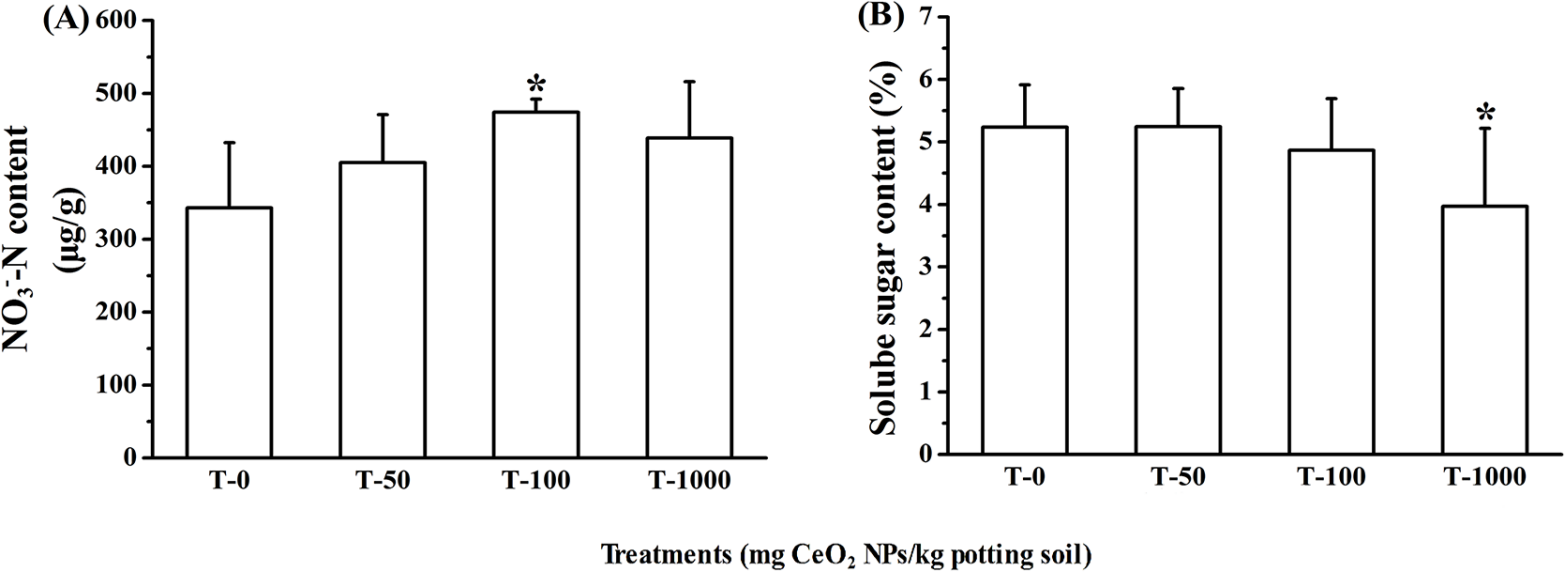
NO_3^-^_-N (A) and soluble sugar (B) contents content in the leaves Error bars stand for standard errors. Bar with this asterisk (*) symbol shows statistically significant differences at *p*≤0.05, n = 8.

Physiological effects of CeO_2_ NPs on nitrate and soluble sugar contents in lettuce plants were shown in Fig 4. In 100 mg·kg^-1^ group, CeO_2_ NPs significantly increased the nitrate content by 38.2%, compared with the control. Nitrate is one of the major N sources for high plants; it promotes vegetative development and yield. Recently, there are numerous studies evaluating the impact of CeO_2_ NPs on quality of plants, but few reports with nitrate. The finding explained why CeO_2_ NPs promoting lettuce growth at certain concentrations. However, excessive nitrogen could influence the quality of vegetable.[32] Nitrate itself is relatively non-toxic but its metabolites may produce a number of negative health effects.

Sugar is one of the three major nutrients of plant. CeO_2_ NPs had a significantly inhibitory effect on the soluble sugar content of at the concentration of 1000 mg·kg^-1^. CeO_2_ NPs at a concentration of 50 mg·kg^-1^ had no impacts on biomass, chlorophyll content, protein, enzyme activity; probably due to the fact that CeO_2_ NPs were absorbed by potting soil, leading to reduction of Ce bioavailability. It is not surprising to see that at 100 mg·kg^-1^, CeO_2_ NPs stimulated the lettuce growth, which is in consistent with an earlier study that Yuan et al. reported a fertilizer containing Ce increased root elongation of rice seedlings, attributed to the Ce^3+^.[33]

### Antioxidant defense system in roots and shoots

**Fig 5:**
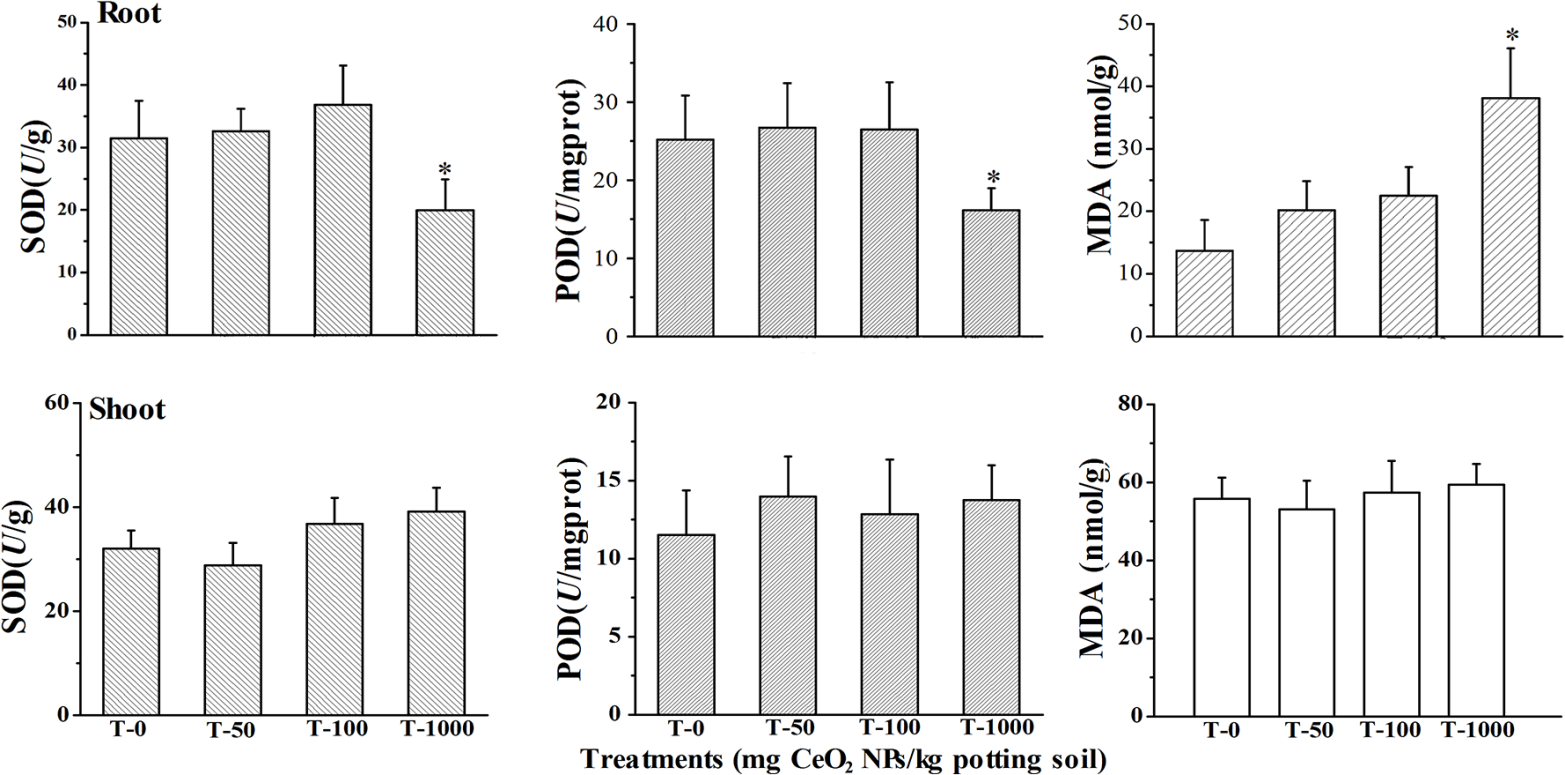
SOD and POD activities and MDA levels in the roots and shoots of of lettuce plants Error bars stand for standard errors. Bar with this asterisk (*) symbol shows statistically significant differences at *p*≤0.05.

The plant cells have antioxidant defense system composed of enzymes, such as SOD and POD that are known to be involved in the detoxification of H_2_O_2_ by converting the H_2_O_2_ to water and oxygen. These root enzymes were differently affected by the concentrations of CeO_2_ NPs used in this study. As seen in Fig 5A, SOD and POD activities of lettuce roots were slightly up-regulated by 50 and 100 mg kg^-1^ CeO_2_ NPs exposure, but significantly down-regulated at 1000 mg kg^-1^. Additionally, the high concentrations of CeO_2_ NPs led to high levels of MDA. The antioxidant defense system of plant cells is composed of SOD, POD and other enzymes, the MDA is a product of degradation of cell membrane components damage of free radicals.^15^ And it can be accepted that the toxicity of CeO_2_ NPs may be caused by excessive production of reactive oxygen species (ROS). These excessive ROS would be cleared by antioxidant enzymes, such as SOD and POD. But if the ROS had not been cleared timely, they would have some damages to the plant cells membrane, also damage the antioxidant defense system, means the increase of MDA levels and the decrease of SOD and POD contents.

Similar to the roots, SOD and POD activities in the shoots were also only slightly up-regulated at all test concentrations of CeO_2_ NPs (Fig 5B). But MDA levels remained constant. These suggested that the CeO_2_ NPs had little impacts on the physiology of the shoots.

## Conclusion

In this research, we investigated the fate and phytotoxicity of CeO_2_ NPs amended in soil. Results show that CeO_2_ NPs were uptake by lettuce plant, and had a positive effect on plant growth at 100 mg kg^-1^, whereas inhibited plant growth at 1000 mg kg^-1^. Meantime, SOD and POD activities and MDA levels was also disrupted by 1000 mg kg^-1^ treatment. The toxicity was probably attributed to the biotransformation of CeO_2_ NPs and the high sensitivity of *Lactuca* plants to the released Ce^3+^ ions.

## Supporting Information

S1 Fig TEM image of sigma CeO_2_ NPs. (JPG)

S2 Fig Chlorophyll contents in the leaves of lettuce plants grown for 30 days in potting soil, treated with 0 (control)-1000 mg kg^-1^ CeO_2_ NPs. (TIF)

S3 Fig Root and shoot protein of lettuce plants grown for 30 days in potting soil treated with 0 (control)-1000 mg kg^-1^ CeO_2_ NPs. Error bars stand for standard errors. Bar with this asterisk (*) symbol shows statistically significant differences at p≤0.05.
(TIF)

S1 Data The data for all of the experiments. (XLSX)
