# Supplementary figures and images for: Fate and Phytotoxicity of CeO2 Nanoparticles on Lettuce Cultured in the Potting Soil Environment
Source: PLoS One. 2015 Aug 28;10(8):e0134261. doi: 10.1371/journal.pone.0134261 (PMC4552829; doi:10.1371/journal.pone.0134261)

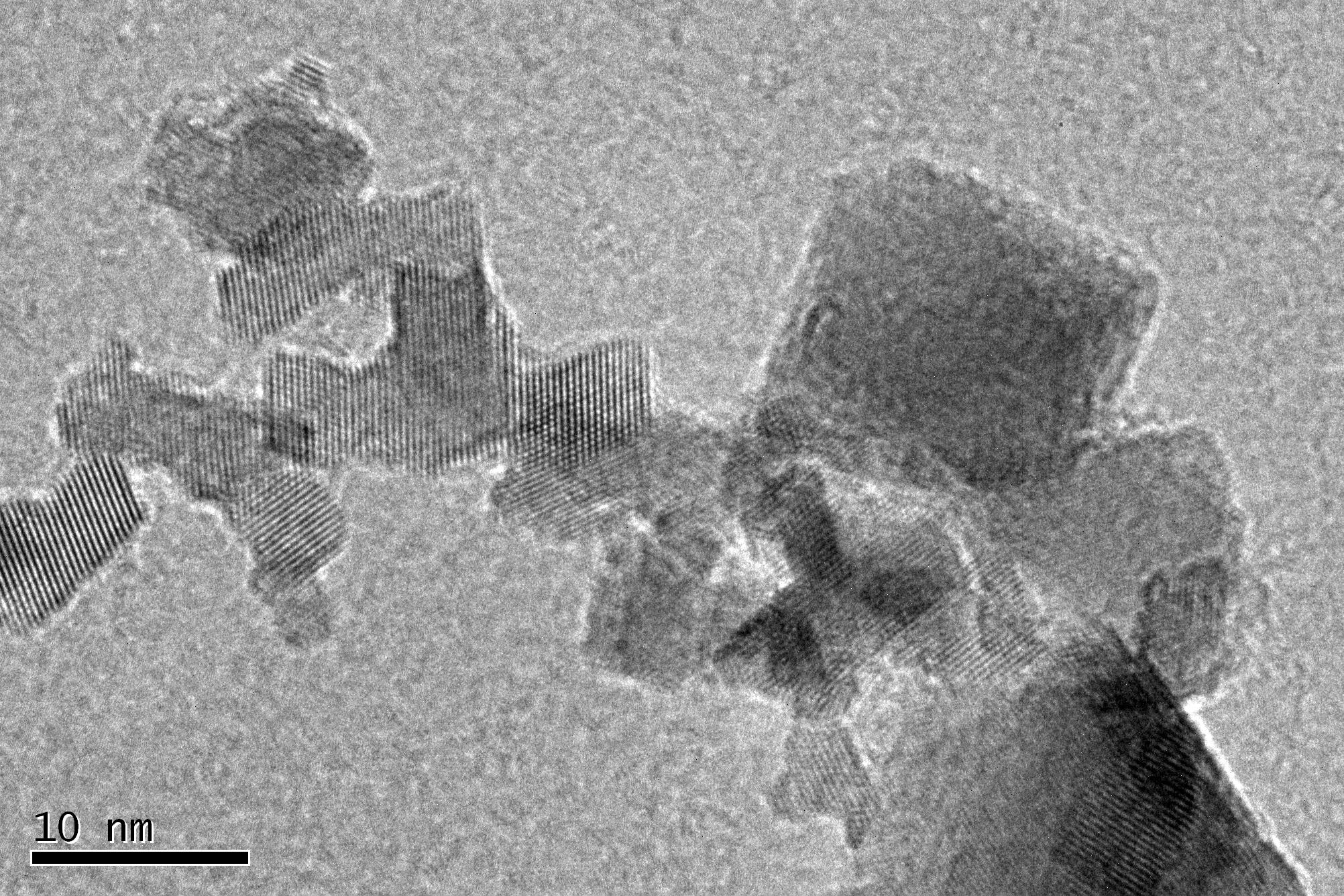

Supplement: S1 Fig — (JPG) [file pone.0134261.s001.jpg]

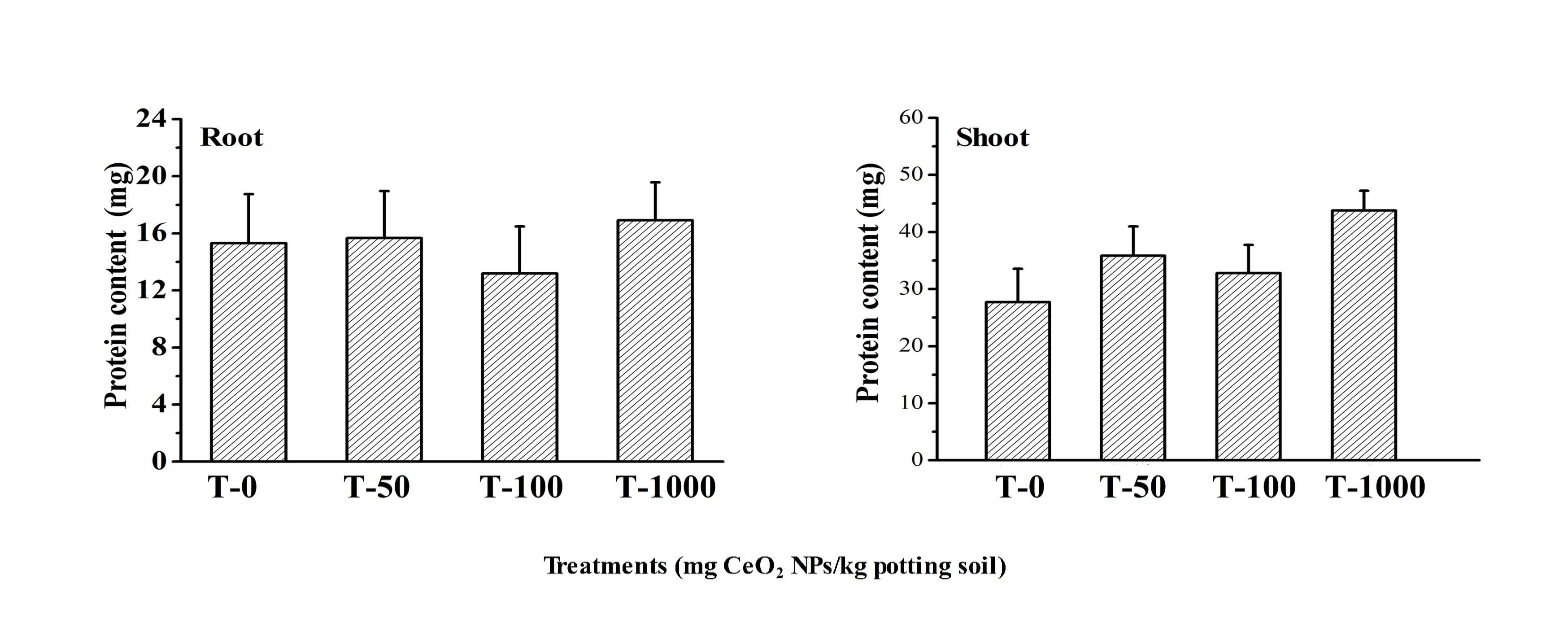

Supplement: S3 Fig — Error bars stand for standard errors. Bar with this asterisk (*) symbol shows statistically significant differences at p≤0.05. (TIF) [file pone.0134261.s003.tif]
